# Reproductive Suicide: Similar Mechanisms of Aging in *C. elegans* and Pacific Salmon

**DOI:** 10.3389/fcell.2021.688788

**Published:** 2021-08-27

**Authors:** David Gems, Carina C. Kern, Joseph Nour, Marina Ezcurra

**Affiliations:** ^1^Institute of Healthy Ageing, Research Department of Genetics, Evolution and Environment, University College London, London, United Kingdom; ^2^School of Biosciences, University of Kent, Canterbury, United Kingdom

**Keywords:** aging, *C. elegans*, programmatic aging, reproductive death, semelparity, senescent pathology

## Abstract

In some species of salmon, reproductive maturity triggers the development of massive pathology resulting from reproductive effort, leading to rapid post-reproductive death. Such reproductive death, which occurs in many semelparous organisms (with a single bout of reproduction), can be prevented by blocking reproductive maturation, and this can increase lifespan dramatically. Reproductive death is often viewed as distinct from senescence in iteroparous organisms (with multiple bouts of reproduction) such as humans. Here we review the evidence that reproductive death occurs in *C. elegans* and discuss what this means for its use as a model organism to study aging. Inhibiting insulin/IGF-1 signaling and germline removal suppresses reproductive death and greatly extends lifespan in *C. elegans*, but can also extend lifespan to a small extent in iteroparous organisms. We argue that mechanisms of senescence operative in reproductive death exist in a less catastrophic form in iteroparous organisms, particularly those that involve costly resource reallocation, and exhibit endocrine-regulated plasticity. Thus, mechanisms of senescence in semelparous organisms (including plants) and iteroparous ones form an etiological continuum. Therefore understanding mechanisms of reproductive death in *C. elegans* can teach us about some mechanisms of senescence that are operative in iteroparous organisms.

## Introduction: *C. Elegans* as a Model for Understanding Human Aging

In its later stages, aging (senescence) manifests as an array of pathologies whose large number and complexity makes understanding its initial causes difficult. For this reason, simple animal models with the possibility of fully understanding senescence, such as *Caenorhabditis elegans*, are invaluable. Studies of this free-living nematode have yielded many insights into biological mechanisms of aging. These include acceleration of aging by insulin/IGF-1 signaling (IIS), germline signaling, mitochondrial function, loss of protein folding homeostasis, but not oxidative damage, and modulation of aging by steroid hormones and epigenetic changes (Greer et al., 2010; Kenyon, 2010; Van Raamsdonk and Hekimi, 2010; Antebi, 2013; Labbadia and Morimoto, 2014; Munkácsy and Rea, 2014).

The extent to which the primary causes of aging in *C. elegans* are the same or different to those in humans will only become clear once both are fully understood. However, it is already evident that *C. elegans* and mammals share some but not all senescent etiologies. For example, in mammals stem cell exhaustion (Shaw et al., 2010; Conboy and Rando, 2012) and accumulation of senescent cells (van Deursen, 2014) (*sensu* Hayflick; note that there are two distinct meanings of the word *senescence*) contribute to senescence in the broad sense. By contrast, in adult *C. elegans* somatic cells are post-mitotic, and cellular senescence (*sensu* Hayflick) does not seem to occur. By contrast, interventions reducing insulin/IGF-1 or mTOR (mechanistic target of rapamycin) signaling or supporting protein folding homeostasis protect against aging in *C. elegans* and mammals (Zhang and Cuervo, 2008; Kenyon, 2010; Labbadia and Morimoto, 2014). Moreover, interventions causing loss of antioxidant defense or mitochondrial impairment which cause death in mammals can increase lifespan in *C. elegans* (Rea, 2005; Van Raamsdonk and Hekimi, 2009).

We recently proposed that two forms of programmatic aging are major determinants of *C. elegans* lifespan: adaptive death, which promotes fitness (i.e., provides a fitness benefit) in a manner similar to apoptosis (Lohr et al., 2019; Galimov and Gems, 2020, 2021), and reproductive death (Kern et al., 2020, 2021). In this essay, we explore further the possibility that *C. elegans* undergoes semelparous reproductive death by comparing it with other organisms known to undergo reproductive death. We then discuss the implications of reproductive death in *C. elegans*, and argue that some mechanisms of senescence are operative in both semelparous and iteroparous organisms.

## Antagonistic Pleiotropy and Programmatic Mechanisms as Conserved Causes of Aging

The predominant causes of aging are the ultimate, evolutionary processes that generate proximate biological mechanisms that cause senescent pathology (Flatt and Schmidt, 2009). One evolutionary cause of aging that is shared between *C. elegans* and humans is antagonistic pleiotropy (AP). Here gene variants that provide a fitness benefit in early life can be favored by natural selection, even where as a side effect they promote pathology in later life (Williams, 1957). How AP acts in terms of proximate mechanisms to cause aging remains unclear.

A traditional interpretation is that trade-offs promoting senescence involve physiological costs in terms of reduced allocation of resources to somatic maintenance (Kirkwood and Rose, 1991), but there are also other possibilities. For example, a different type of AP mechanism altogether, suggested in a hypothetical example by George Williams himself, is continued wild-type gene action in late life with pathogenic effects (Williams, 1957). A more recent elaboration of this idea, drawn in particular from the effects of mTOR, is that late-life action of regulators of growth and reproduction results in futile and pathogenic execution of complex biological programs (de Magalhães and Church, 2005; Blagosklonny, 2006). Because the term *program* implies the presence of a function, while such late-life action is futile, Blagosklonny introduced the term *quasi-program*; in other words, programmed in the mechanistic sense but not the adaptive sense (Galimov et al., 2019). More broadly, one may accurately describe proximate mechanisms of this type as *programmatic* (de Magalhães and Church, 2005; Maklakov and Chapman, 2019). As a primary mechanism of aging, this form of AP is distinct from damage accumulation and, in the case of IIS/mTOR for example, results not from a passive loss of function (or wearing out), but rather active gene function, or *hyperfunction* (Blagosklonny, 2008) (see Glossary for definition of key terms).

**FIGURE 1:**
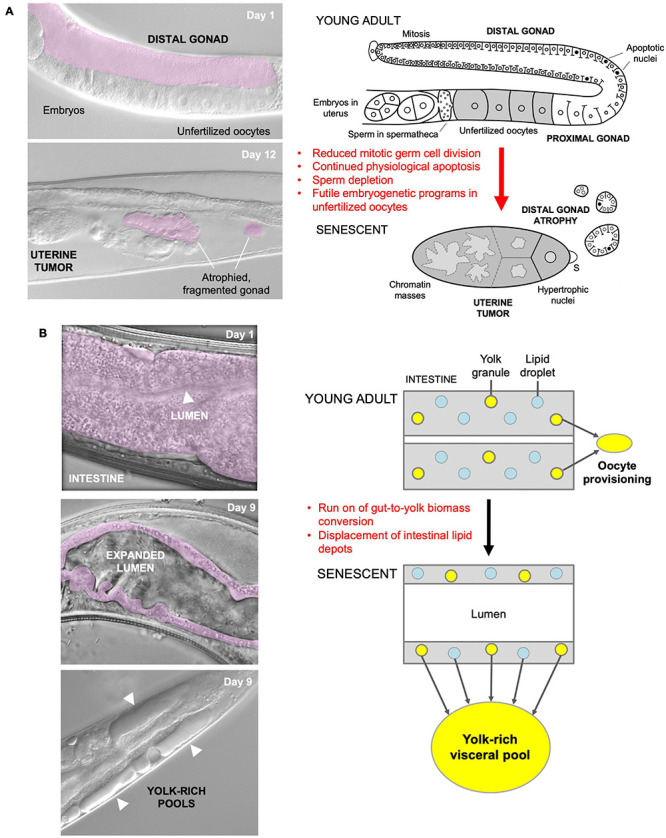
Quasi-programs as a cause of *C. elegans* hermaphrodite senescence. **(A)** Distal gonad atrophy and teratoma-like uterine tumor formation. Left: appearance of pathologies under Nomarski microscopy; distal gonad marked in pink. Right: proposed pathophysiology involving quasi-programs. In the young adult oocytes are generated by proliferation of mitotic germline stem cells which then enter meiosis, and then in most cases undergo physiological apoptosis (PA) to generate cytoplasm to fill expanding oocytes (Gumienny et al., 1999; Jaramillo-Lambert et al., 2007; Wolke et al., 2007). Subsequently, declining stem cell division (conceivably adaptive) (Kocsisova et al., 2019) and run-on of PA promotes distal gonad atrophy and fragmentation (de la Guardia et al., 2016). Unfertilized oocytes fail to complete meiosis, enter the uterus and develop into teratoma-like tumors containing massively polyploid chromatin masses (Golden et al., 2007) which appears to result, as in mammalian ovarian teratomas, from embryonic quasi-programs (Wang et al., 2018a,b). **(B)** Left, intestinal atrophy and yolk-rich visceral pool accumulation. Right, hypothesis for etiology of both pathologies: a vitellogenic quasi-program, where remobilization of intestinal biomass into yolk continues in a futile fashion (Ezcurra et al., 2018; Benedetto and Gems, 2019).

Our recent studies of several major *C. elegans* senescent pathologies imply that they originate predominantly from hyperfunction rather than molecular damage (Gems and de la Guardia, 2013; de la Guardia et al., 2016; Ezcurra et al., 2018; Wang et al., 2018b; Sornda et al., 2019). For example, physiological apoptosis (PA) in the hermaphrodite germline supports nascent oocyte growth, and apparently futile run-on of PA contributes to gonad atrophy and fragmentation (Figure 1A; de la Guardia et al., 2016). In another example, activation of embryogenetic functions in unfertilized oocytes in the uterus leads to extreme polyploidy, cellular hypertrophy and teratoma-like tumors (Figure 1A; McGee et al., 2012; Wang et al., 2018a, b). In both cases, quasi-programs promoted by wild-type gene action contribute to the development of major senescent pathology.

As a further example, during hermaphrodite aging large pools of material that appears oily when viewed using Nomarski microscopy accumulate in the body cavity (Figure 1B), and contain vitellogenin (yolk protein) and lipid (Garigan et al., 2002; Herndon et al., 2002; McGee et al., 2011; Yi et al., 2014; Chen et al., 2016; Ezcurra et al., 2018). Such pseudocoelomic lipoprotein pools (PLPs) represent a form of senescent steatosis (Palikaras et al., 2017; Ezcurra et al., 2018). Moreover, levels of vitellogenins increase dramatically, reaching up to sevenfold of that seen in young adults (Depina et al., 2011; Ezcurra et al., 2018; Sornda et al., 2019). Given that this accumulation occurs in post-reproductive hermaphrodites, it appears to be the result of futile, open faucet-type run-on of yolk synthesis, or a vitellogenic quasi-program (Herndon et al., 2002; Gems and de la Guardia, 2013; Ezcurra et al., 2018).

The *C. elegans* intestine is the largest somatic organ and serves multiple functions, including those played by the liver and adipose tissue in vertebrates (McGhee, 2007). It is a site of action of genes affecting lifespan (Lin et al., 2001; Libina et al., 2003; Venz et al., 2021). During aging in *C. elegans* hermaphrodites, the intestine undergoes major atrophy, losing most of its volume (Figure 1B; Garigan et al., 2002; McGee et al., 2011; Ezcurra et al., 2018). The intestine is the site of yolk synthesis for oocyte provision (Kimble and Sharrock, 1983), and consumption of intestinal biomass to support continued yolk export is a cause of intestinal atrophy (Ezcurra et al., 2018; Sornda et al., 2019). Loss of function of genes supporting autophagy inhibits both intestinal atrophy and PLP accumulation, suggesting that autophagy facilitates gut-to-yolk biomass conversion, and that futile run-on of vitellogenesis promotes intestinal atrophy (Ezcurra et al., 2018; Sornda et al., 2019).

These proximate, pathogenetic mechanisms are distinct from molecular damage accumulation, traditionally viewed as the predominant cause of aging; however, this does not rule out a contributory role for molecular damage in aging in general.

## Yolk Venting Suggests That *C. Elegans* Could Be Semelparous

**FIGURE 2:**
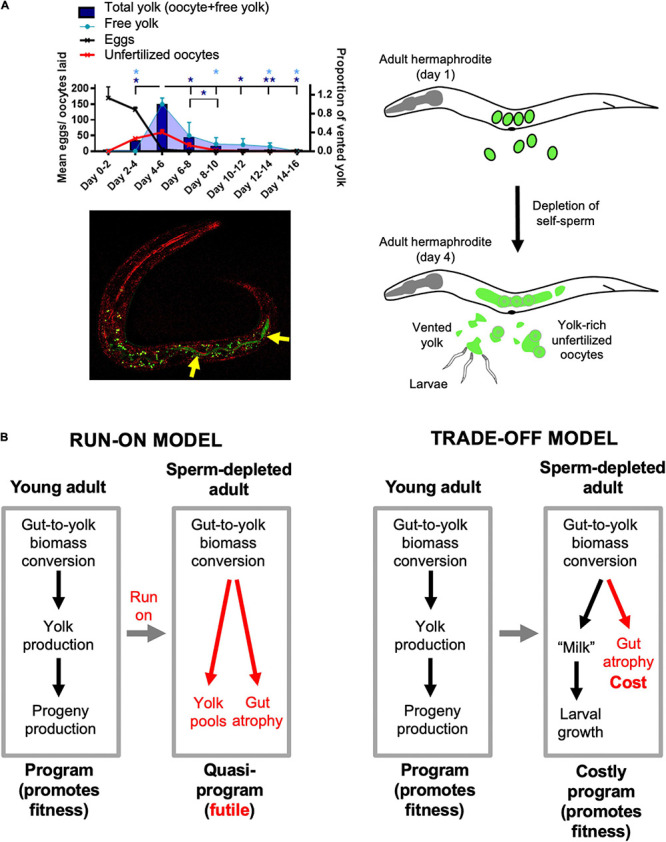
Lactation by *C. elegans* hermaphrodites, and its implications. **(A)** Trophallaxis (“milk” provision) by *C. elegans*. Top left: schedule of production of eggs, unfertilized oocytes and vented yolk by wild-type *C. elegans* hermaphrodites (20°C); *p < 0.05, **p < 0.01, one-way ANOVA. Bottom left: L1 larva with ingested yolk in intestinal lumen (reproduced from Kern et al., 2021). Green: yolk marked with VIT-2:GFP (arrows); green dots are autofluorescent gut granules. Red, reflective confocal microscopy to highlight intestinal lumen (intestinal cell apices). Right: scheme showing transition from egg laying to yolk (milk) venting after hermaphrodite self-sperm depletion. **(B)** Implications: two interpretations of origins of intestinal atrophy. Left: After sperm depletion the program for yolk synthesis runs on to become a futile quasi-program (Ezcurra et al., 2018). Right: after sperm depletion the program for yolk production becomes a costly program supporting lactation (Kern et al., 2021).

The interpretation of late-life yolk production as quasi-programmed is based on the reasonable assumption that it is futile, but is it really? Could later yolk accumulation somehow promote fitness? Our recent study of the phenomenon of yolk venting supports the latter possibility (Kern et al., 2021). Beginning at the end of egg laying, hermaphrodites vent substantial amounts of liquid rich in vitellogenins and lipid through the vulva and into their local vicinity. Notably, consumption by larvae of this vented yolky substance, present either as free pools or within unfertilized oocytes, can promote larval growth and increase fertility (Kern et al., 2021). This suggests a later function for vented yolky fluid similar to that of milk (we suggest the term *yolk milk*). Feeding of milk-like fluid by mothers to offspring has been observed before in various other invertebrates, such as the Pacific beetle cockroach, *Diploptera punctata* (Marchal et al., 2013) and the tsetse fly (*Glossina* spp.) (Benoit et al., 2015). Such behavior exemplifies the wider phenomenon of trophallaxis, the social transfer of nutrient fluids between individuals, particularly in the context of parental care. Trophallaxis also encompasses fluid exchanges between social insects and mammalian nursing (LeBoeuf, 2017). In *C. elegans*, mutation of the *daf-2* insulin/IGF-1 receptor, which greatly extends lifespan, also suppresses venting of both yolk and unfertilized oocytes (Kenyon et al., 1993; Gems et al., 1998; Kern et al., 2021). Function as a vector for trophallactic fluid (Figure 2A) could provide an answer to the long-standing mystery of why adult hermaphrodites lay more than their own volume in unfertilized oocytes (Ward and Carrel, 1979).

If late-life yolk production provides a fitness benefit, then yolk steatosis and intestinal atrophy are not the result of a vitellogenic quasi-program. Instead, intestinal atrophy results from a life history trade-off involving physiological costs (Figure 2B). As previously defined, physiological costs can be either direct (e.g., the energy or nutrient requirements of reproduction) or indirect (Zera and Harshman, 2001; Speakman, 2008). Indirect costs include consequential costs, where harm occurs unavoidably as a consequence of the reproductive event, for example bone loss in mammals due to calcium remobilization during lactation (Speakman, 2008). In that example and in intestinal involution to support yolk milk production in *C. elegans* (Ezcurra et al., 2018), an active, programmed process of resource reallocation promotes fitness; however, intestinal atrophy itself is a pathological side effect and does not promote fitness. A further, formal possibility here is that gut-to-yolk resource reallocation includes resources diverted from cellular processes that protect against molecular damage.

The existence of yolk milk venting as a means of resource transfer from post-reproductive mothers to larval kin could also resolve another puzzle, relating to the overall pattern of senescent pathogenesis in *C. elegans*. In humans, age-related diseases appear late in life after an extended period of optimal health (Niccoli and Partridge, 2012). However, in *C. elegans* hermaphrodites, development of senescent pathologies begins within days of reproductive maturity (Ezcurra et al., 2018), and involves a level of destructive severity (including massive organ hypertrophy, atrophy, and disintegration) that is not typical of senescence in higher animals (Garigan et al., 2002; Herndon et al., 2002; McGee et al., 2011, 2012; de la Guardia et al., 2016). By contrast, in wild-type males, these pathologies are not seen (de la Guardia et al., 2016; Ezcurra et al., 2018). This pattern of rapid and severe pathological change affecting organs linked to reproduction [the nervous system is relatively well preserved in aging *C. elegans* (Herndon et al., 2002)] is reminiscent of semelparous organisms that undergo programmed reproductive death. Previously, the apparent absence of any fitness benefit to which these destructive changes could be linked as a cost argued against the idea that *C. elegans* is semelparous. However, with the discovery of “lactation” in *C. elegans*, it now appears more likely that this organism is semelparous. To explore this possibility, let us next consider semelparity in more detail.

## Semelparity and Reproductive Death

*Comparer, c’est comprendre.* Charles de Gaulle

Life histories may be broadly classified according to reproductive schedule, where semelparous species reproduce once and iteroparous species more than once (Cole, 1954; Finch, 1990b); but more precisely, semelparity and iteroparity represent two ends of a continuum of parity (Hughes, 2017). Reproduction in semelparous species can lead to rapid, post-reproductive death (reproductive death) by various mechanisms, usually coupled to very high levels of reproductive effort and investment which leads rapidly to severe pathology (Finch, 1990b). Though semelparous organisms do not necessarily undergo reproductive death, the term *semelparous* is sometimes used to denote semelparity with reproductive death; for convenience, we will often follow that usage here. In many semelparous organisms, rapid senescence is triggered by sexual maturation and under hormonal control. This form of reproductive death can be prevented, for example by surgical removal of organs that direct physiological changes that lead to death or by removing environmental cues, and this can result in increases in lifespan of a large magnitude (as detailed below).

**FIGURE 3:**
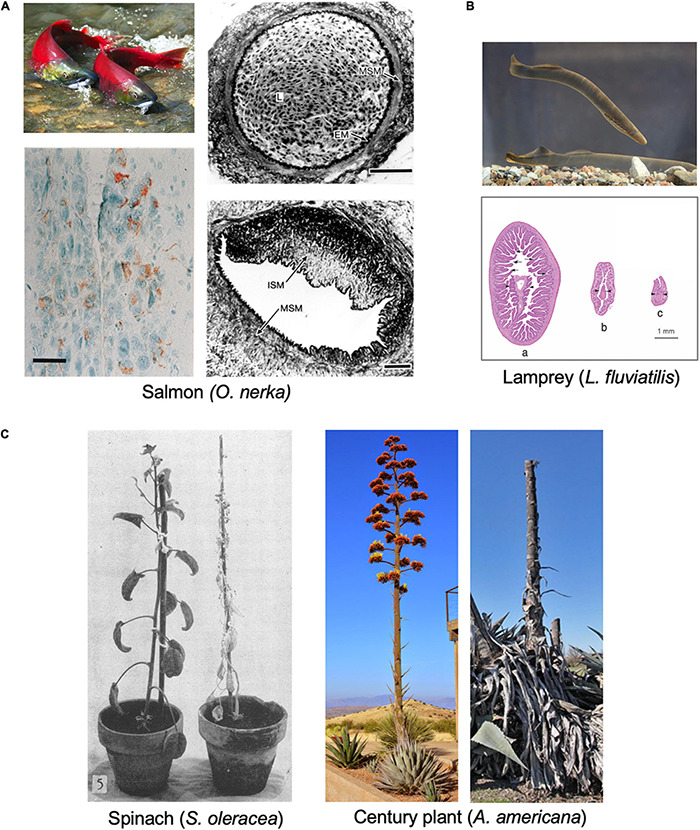
Examples of semelparous organisms and their senescent pathologies. **(A)** Pacific salmon *O. nerka*. Top left: sexually mature adults (photo courtesy of Georgia Strait Alliance, www.georgiastrait.org© Olga Vasik—Adobe Stock). Bottom left: Immunoreactivity to Aβ_1_–_42_ antibody in the brain of spawning kokanee salmon (Maldonado et al., 2000), c.f. amyloid plaques associated with Alzheimer’s disease. Bar, 20 μm. Right: cross section of normal coronary artery (top); L, lumen, filled with nucleated red blood cells; MSM, medial layer of vascular smooth muscle; EM, elastic membrane; ISM; or (bottom) from mature adult with severe arteriosclerotic lesion, containing mainly intimal smooth muscle cells (ISM) (Farrell, 2002). Bars, 50 μm. **(B)** Lamprey (genus *Lampetra*). Top, European river lamprey (*L. fluviatilis*) (photo by Tiit Hunt, distributed under a CC BY-SA 3.0 license). Bottom: stages of intestinal atrophy during spawning in *L. japonica*. Diameters of (a) 3.9 mm, (b) 1.5 mm (b), and (c) 1 mm. Arrows, intestinal villi; arrowheads, typhosole (internal intestinal fold) (Higashi et al., 2005). **(C)** Examples of reproductive death in semelparous plants. Left, *Spinacia oleracea* 45 days after full bloom; reproductive death (right) has been suppressed by flower removal (left) (Leopold et al., 1959) (© American Society of Plant Biologists, reprinted with permission). Right, *Agave americana* during and after flowering (photos by Gerhard Bock, reproduced with permission). Century plants typically live 10–30 years, and death follows rapidly after a single massive reproductive event.

The biology of animal semelparity has been explored in more detail in vertebrates than invertebrates. Semelparity in vertebrates is rare, but found in some fish (e.g., salmon, lampreys, eels), and a few reptiles (e.g., the aspic viper) (Bonnet, 2011) and marsupial mammals. Semelparity in Pacific salmon such as *Oncorhynchus nerka* has been studied in some detail. Semelparous salmon are usually anadromous, migrating from the sea to spawn in fresh water. When swimming up river they undergo marked anatomical changes where testes and ovaries grow dramatically, plasma vitellogenin levels rise (von der Decken, 1992), and males develop secondary sexual characteristics, including growth of the beak to form the hook, and hump development (Quinn and Foote, 1994; Figure 3A). These changes are triggered by gonadal steroids, leading to increased corticosteroid production (Hane and Robertson, 1959; Mcquillan et al., 2003), which mobilizes energy to support reproduction but also impairs immune defense mechanisms, in a manner that resembles Cushing’s disease in humans (hyper-adrenocorticism). As in *C. elegans* hermaphrodites, a range of severe, deteriorative pathologies rapidly develop, here affecting the liver, kidney, spleen, heart, thymus, and digestive tract (Robertson et al., 1961; Finch, 1990b). Death occurs a week or two after spawning (Carruth et al., 2002).

Reproductive death is also seen in lampreys, jawless fish of the class Agnatha, such as the European river lamprey *Lampetra fluviatilis* (Figure 3B). Lampreys pass through larval and non-reproductive juvenile stages of variable duration before undergoing sexual maturation and spawning, usually after around 4–8 years. Prior to spawning in fresh water they cease feeding, and before and during sexual maturation undergo major anatomical changes including atrophy of many somatic organs, such as the body wall (including muscle), intestine and liver (but not the heart), and organism-wide loss of protein, glycogen, and fat, which supports both gonadal growth (including vitellogenesis by the liver) and swimming (Bentley and Follett, 1965; Larsen, 1969, 1980; Mewes et al., 2002). Atrophy of the intestine is particularly marked (Larsen, 1965, 1969; Higashi et al., 2005; Figure 3B), reminiscent of *C. elegans* hermaphrodites (Ezcurra et al., 2018), but this occurs prior to sexual maturation, where the main source of remobilized resources is the body wall (Larsen, 1980). Death occurs shortly after spawning (a few days or weeks) (Larsen, 1980). Intestinal atrophy during sexual maturation has also been documented in eel species (genus *Anguila*) (Pankhurst and Sorensen, 1984).

A number of dasyurid marsupials of the genera *Antechinus*, *Phascogale*, and *Dasykaluta* exhibit reproductive death (Braithwaite and Lee, 1979; Hayes et al., 2019). For example, males of the mouse-like brown antechinus *A. stuartii* enter the breeding season at around the end of their first year of life, and most die within 2–3 weeks of reproductive maturity (Woolley, 1966). As in semelparous salmon, a major driver of pathology is hypercorticism associated with adrenal hyperplasia, which causes the males to become ill and die, e.g., due to infection and gastrointestinal hemorrhage (Barker et al., 1978; Bradley et al., 1980). Reproductive death is seen particularly in plants, as detailed below (Figure 3C).

A common feature of semelparous species is an extended pre-reproductive stage, with death following rapidly after reproductive maturation. For example, eels of the genus *Anguilla* typically spawn and die at 6–12 years of age (Tesch, 1977), and the bamboo *Phyllostachys bambusoides* flowers and dies after as much as 120 years (Janzen, 1976; Soderstrom and Calderon, 1979). As previously noted (Finch, 1990b, p. 118), *C. elegans* shows this pattern: diapausal dauer larvae can survive for up to 90 days, whereas after recovery from dauer and attainment of adulthood, death occurs within 2–3 weeks (Klass and Hirsh, 1976; Klass, 1977).

In conclusion, the pattern of pathological anatomical change seen *C. elegans* hermaphrodites resembles that seen in reproductive death, particularly in semelparous fish. Next we explore the similarities between *C. elegans* and semelparous organisms in terms of the possible proximate mechanisms of aging involved.

## Destructive Resource Reallocation in Reproductive Death

**FIGURE 4:**
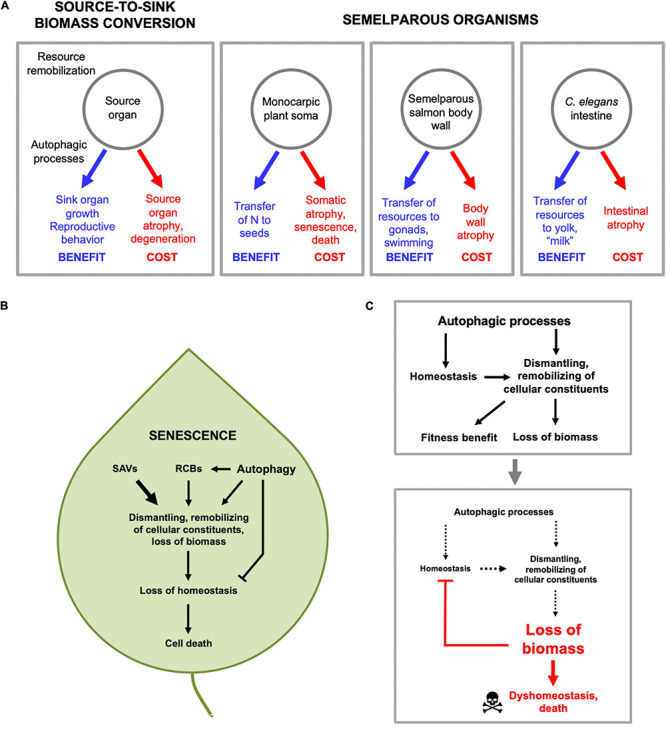
Source-to-sink biomass conversion and physiological costs that cause pathology. **(A)** General form of source-to-sink biomass conversion (left) and three examples. In each case remobilization of resources lead to fitness benefits by supporting reproductive processes, but leads to atrophy and eventual pathology in source organs. **(B)** Autophagic processes and senescence in plants. Material from other organelles, particularly chloroplasts, is transported to the vacuole in several ways, including autophagosomes. First, via autophagosomes, double membrane-bound vesicles as found in animal and fungal autophagy pathways (Marshall and Vierstra, 2018). Second, via double membrane-bound rubisco-containing bodies (RCBs; rubisco is the most abundant stromal protein in chloroplasts) which contain fragments of chloroplast proteins (Chiba et al., 2003), and whose transport to the vacuole is dependent on genes of the autophagy pathway (Ishida et al., 2008; Wada et al., 2009). Third, via senescence-associated vacuoles (SAVs) which are single membrane bound and which, unlike autophagosomes, contain high levels of protease activity (Martinez et al., 2008). **(C)** Autophagic processes protect in order to destroy (demolition engineer principle). A hypothesis based on recent progress in understanding the role of autophagy in plant leaf senescence (Avila-Ospina et al., 2014) (with thanks to Prof. Céline Masclaux-Daubresse). Top: by maintaining homeostasis during the systematic destruction of the cell, autophagic processes aid in its destruction. Bottom: eventually the cell is dismantled to the point that even autophagic processes cannot be sustained, and homeostasis collapses, leading to death.

Our working hypothesis is that *C. elegans* reproductive death results, at least partly, from the costs of consequential indirect physiological trade-offs, including one in which intestinal biomass is consumed to generate trophallactic fluid (yolk milk) that nourishes larval kin (Speakman, 2008; Ezcurra et al., 2018; Kern et al., 2021). Broadly, this is a type of process where biological structures at one site (the source) are broken down and converted into structures at another location, or into activity (the sink). As has been said: “The massive translocation of resources at the time of reproduction is fundamental to the biology of semelparous species” (Young and Augspurger, 1991). While providing a fitness benefit at the sink, source organs can be impaired, e.g., due to atrophy (Figure 4A). The nature of reproductive effort supported at the sink can involve increased gonadal development, gamete production (including vitellogenesis) or lactation, or enhanced performance (e.g., courtship, mating). For example, in semelparous salmon, muscle catabolism to generate nutrients supports gonadal and gamete development, and the effort of swimming upstream, but also causes muscle atrophy (von der Decken, 1992). Similarly, in eels and lampreys atrophy of muscle is coupled to gonad growth and sustained swimming, and in eels skeletal breakdown releases calcium and phosphate for transfer to gonads (Larsen, 1980; Freese et al., 2019). Again, during their brief breeding season male *A. stuartii* cease feeding, and glucose availability is increased by gluconeogenesis promoted by elevated plasma corticosteroid levels, which both provides energy to support their extended copulatory exertions (increased performance; *A. stuartii* will copulate for up to 8 h continuously) and causes lethal immune deficiency (Naylor et al., 2008).

### The Role of Autophagy in Source-to-Sink Biomass Conversion

Source-to-sink biomass conversion implies the occurrence of bulk autolysis of biomass in the source tissue. This suggests a role of enzymatic degradation, which usually occurs within acidic compartments within the cell, including lysosomes in animals, and the vacuole in fungi and plants (Figure 4A). In animals, the major, regulated intracellular mechanism of bulk autolysis is autophagy (specifically macroautophagy). In *C. elegans*, inhibition of autophagy inhibits both intestinal atrophy and yolk steatosis (Ezcurra et al., 2018). The implied role of autophagy as a promoter of senescent pathology is somewhat unexpected given previous evidence that autophagy is important in maintaining homeostasis and protecting against senescent decline (Gelino and Hansen, 2012). Plausibly, physiological costs due to biomass conversion are more severe in semelparous than iteroparous organisms, such that a major role for autophagic processes in pathogenesis is a special feature of semelparity. Very little is known about the role of autophagy in reproductive death in animals. In lampreys, breakdown of intestinal biomass occurs in part in the stellate cells beneath the intestinal epithelium. In *L. japonica* there is some evidence that biomass breakdown (visible as loss of collagen fibrils) occurs by a process of phagocytosis and lysosomal proteolysis (Higashi et al., 2005). Intestinal atrophy in lampreys occurs largely prior to vitellogenesis, which occurs in the liver (Larsen, 1980), so lampreys differ from *C. elegans* here.

### Destructive Resource Reallocation and Senescence in Plants

Much more is known about the biology of source-to-sink biomass conversion in plants, in the context of semelparity (in plants, monocarpy), and also leaf senescence (Young and Augspurger, 1991; Davies and Gan, 2012; Avila-Ospina et al., 2014). One reason is that semelparity is much more common among plants than animals. Another is that understanding the biology of biomass conversion is useful for crop improvement. This knowledge includes a detailed understanding of the proteolytic machinery involved in autolysis (including autophagy) in source tissues that provides useful insight into semelparous pathophysiology.

In deciduous trees in autumn, leaf senescence occurs during which leaf biomass is broken down and remobilized (particularly nitrogen), and transported via the phloem to support tree survival, resulting in leaf death. In many monocarpic angiosperms, the entire soma is broken down during flowering and fruiting, largely to support seed production (Schippers et al., 2015; Diaz-Mendoza et al., 2016). In perennial polycarps the entire plant above ground may die off to support growth and survival of the subterranean bulb. In each case, somatic biomass is transferred from source to sink organs (Davies and Gan, 2012). For example, in wheat and rice grains up to 90% of the nitrogen content is derived from the senescence of somatic tissues (Diaz-Mendoza et al., 2016).

Senescence-associated biomass conversion in plants is driven by action of a variety of proteases acting in different cellular compartments, but the final destination is mainly the large, acidic central vacuole (Avila-Ospina et al., 2014). This is functionally related to the lysosome of animal cells, e.g., as a major site of proteolysis by acid proteases. Material from other organelles, particularly chloroplasts, is transported to the vacuole in several ways, including autophagosomes (Figure 4B). Thus, in plants as in *C. elegans* gut-to-yolk biomass conversion, autophagy and autophagy-related processes promote senescence.

If autophagy promotes plant senescence, then inhibiting autophagy should retard senescence, as seen in *C. elegans* intestinal senescence (Ezcurra et al., 2018; Benedetto and Gems, 2019). The effects of inhibition of autophagy on plant senescence are complex but, interestingly, support the view that autophagy promotes the earlier stages of senescence but protects against its later stages. For example, in *Arabidopsis thaliana* loss of expression of genes encoding proteins involved in autophagy (*atg5, atg9*, or *atg18a*) inhibits the decline with advancing age in amino acid, protein and RNA content in plant rosettes (Guiboileau et al., 2013; Haveì et al., 2018). Loss of *atg5* in plants subjected to mild (but not severe) stress suppresses leaf senescence (Sakuraba et al., 2014). Moreover, *atg* mutants are hypersensitive to N and C starvation, and deficient in N redistribution into seeds, not only in *A. thaliana* but also in maize and rice (Tang and Bassham, 2018). Furthermore, global expression of *atg* genes increases in the later stages of leaf senescence in many plant species, though in *A. thaliana* leaf senescence this occurs after N mobilization is well underway (Avila-Ospina et al., 2014; Tang and Bassham, 2018). Overall, this supports the view that autophagy promotes resource remobilization during senescence leading to loss of somatic biomass.

### Autophagic Processes Maintain Homeostasis While They Destroy the Cell

Overall, studies of autophagy in plant senescence reveal its double-edged role in resource reallocation processes that lead to death. Here autophagy contributes to nutrient recycling and remobilization during leaf senescence, but also helps maintain homeostasis in the cell while it is being dismantled (Avila-Ospina et al., 2014). Thus, in the absence of the classic autophagy pathway, the destructive action of other autophagy-related processes (Figure 4B) would lead more rapidly to leaf dyshomeostasis and death. In other words, autophagy promotes senescence by facilitating resource reallocation, but also protects against it by maintaining homeostasis. However, given that sustaining homeostasis aids resource reallocation, this protective role of autophagy is ultimately destructive (Figure 4C), and analogous to the action of demolition engineers preparing a building for destruction, who work to maintain its structural integrity while stripping out reusable materials. Thus, in this context, autophagy protects in order to destroy.

Leaf senescence provides a lucid illustration of the relationship between the ordered, programmed process by which the plant cell is dismantled, and the resulting homeostatic collapse leading to death. The entire senescence process is pathological (at least with respect to the leaf). Though the leaf loses functionality from the outset of senescence (e.g., photosynthetic), only in its later stages does loss of homeostasis contribute to pathogenesis. The same is the case for many diseases, where the initial impact of etiology may not cause dyshomeostasis, as in early stages of cancer development, or viral infections.

According to the demolition engineer principle outlined above, a general feature of source and sink biomass conversion processes that lead eventually to death is that cells, tissues, and organisms need to remain alive and functioning to be able to efficiently dismantle themselves. For example, during leaf senescence, chloroplasts are broken down early on but mitochondria remain intact and functional until the final stages of senescence (Peterson and Huffaker, 1975; Diaz-Mendoza et al., 2016). Similarly, in *C. elegans*, the intestine and distal gonad undergo atrophy in early adulthood but the nervous system remains intact into late life (Herndon et al., 2002; Ezcurra et al., 2018). Again, in sexually mature lampreys multiple organs (including the intestine and liver) undergo severe atrophy, but the heart is protected (Bentley and Follett, 1965; Larsen, 1980).

The ordered sequential nature of the destruction of organelles, cells and organs in semelparous organisms contrasts with aging in iteroparous organisms, such as mice or humans, where incidence of aging-related diseases varies greatly between individuals (Finch, 1990b; Austad, 2004). For example while mammalian cancers vary in type and incidence, all aging *C. elegans* hermaphrodites develop teratoma-like uterine tumors (Wang et al., 2018b).

### Source-to-Sink Biomass Conversion Is Not Disposable Soma

**FIGURE 5:**
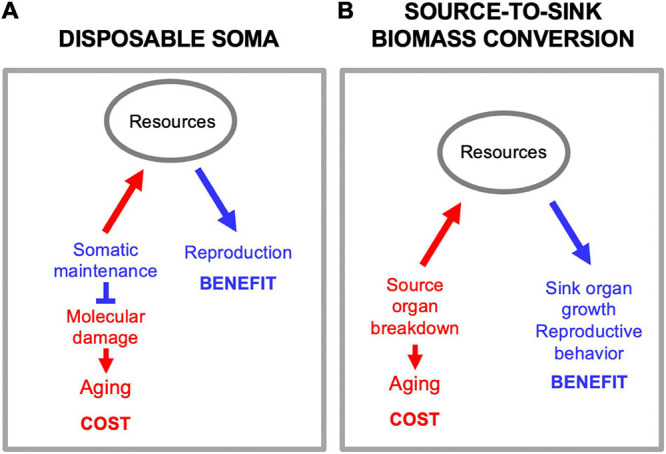
Source-to-sink biomass conversion is not disposable soma. **(A)** Disposable soma. Here a primary cause of aging is stochastic molecular damage accumulation, prevented by somatic maintenance processes. Diversion of resources from somatic maintenance to reproduction provides a reproductive fitness benefit, but allows molecular damage to accumulation, causing aging. **(B)** Source-to-sink biomass conversion. Here a primary cause of aging is programmatic, active self-destruction of organs to release resources for reproduction.

There is a superficial resemblance between biomass conversion and another mechanism proposed to underlie trade-offs between reproduction and lifespan, but they are not the same. The disposable soma theory proposes that stochastic molecular damage causes aging, and that aging rate is determined by the level of resource investment into somatic maintenance mechanisms that prevent that damage (Kirkwood, 1977, 2005; Figure 5A). By contrast, in biomass conversion mechanisms source tissues and organs are actively dismantled in the process of promoting function at the sink (Figure 5B). While it is true that this can involve utilization of somatic tissues in a disposable fashion, this is not the same as the disposable soma theory as set out. The primary etiology is programmatic, not stochastic damage.

## Prevention of Reproductive Death Can Greatly Extend Lifespan

In *C. elegans* hermaphrodites, removal of the germline leaving the somatic gonad intact increases mean lifespan by some 60% (Hsin and Kenyon, 1999). One possibility is that this is due to suppression of reproductive death, which in other semelparous organisms increases lifespan substantially.

### Life Extension by Suppression of Reproductive Death

Reproductive death in semelparous species is actively promoted by hormonal factors, for example corticosteroids in *A. stuartii* and salmon of the genus *Oncorhynchus*, and abscisic acid in monocarpic plants. Blocking production of such factors, e.g., by surgical removal of their source or by behavioral manipulation, can suppress reproductive death. As one would expect, this can cause large increases in lifespan. For example, in the salmon *O. nerka* castration before spawning prevented hypercorticism and increased maximum lifespan from 4.8 to 8.5 years (Robertson, 1961). It has also been suggested that parasitic mollusc larvae can suppress reproductive death and extend lifespan in Atlantic salmon (*Salmo salar*) (Ziuganov, 2005). Moreover, gonadectomy or hypophysectomy (removal of the pituitary gland equivalent) in the lamprey *L. fluviatilis* prior to sexual maturation inhibited body wall mobilization and intestinal atrophy (Larsen, 1974, 1980; Pickering, 1976) and instead of dying shortly after spawning, hypophysectomized animals survived for up to 11 months (Larsen, 1965; reviewed in Larsen, 1980).

In the eel *Anguila anguila* the bulk of pre-adult growth occurs in rivers, and after 6–12 years sexually mature adults make sea runs to spawn and die in the Sargasso Sea (Finch, 1990b). Prevention of the sea run and spawning can increase eel lifespan substantially. For example, one eel kept in a well in Denmark lived for 55 years (at least a 3.5-fold increase in lifespan) (Tesch, 1977), while another maintained in a Swedish aquarium lived for 88 years (at least a sevenfold increase in lifespan) (Vladykov, 1956).

Looking beyond fish, in the octopus *O. hummelincki* removal of the optic gland just after spawning in females increased lifespan measured from onset of egg-laying by up to 5.4-fold (maximum lifespan, from 51 to 277 days) (Wodinsky, 1977). Reproductive death in *A. stuartii* can be prevented either by capture and cage maintenance prior to mating or by castration. If males are captured prior to mating and maintained in the lab they can survive for 3 years or more (Woolley, 1966; Olsen, 1971; Bradley et al., 1980). Removal of reproductive structures can also inhibit senescence in monocarpic plants; for example, removal of flowers prior to pollination increased mean lifespan in soybean plants (*Glycine max*) from 119 to 179 days after sowing (+ 50.4%) (Leopold et al., 1959). The standard view is that, in these instances, extension of lifespan results not from retardation of aging but from prevention of reproductive death (but see below).

Removal of the germline can also increase lifespan in iteroparous species. For example, in *Drosophila subobscura* the *grandchildless* mutation, which causes germline loss, increased life expectancy (from day 10) by 15.1% (Maynard Smith, 1958). In *Drosophila melanogaster* loss of germ cells from late development or early adulthood extended median lifespan in both sexes by 21.0–50.0% (Flatt et al., 2008), but absence of the germline throughout life shortened female lifespan (Barnes et al., 2006). Ovariectomy also increased median lifespan in grasshoppers by 16.3 or 22.7% (Hatle et al., 2008; Drewry et al., 2011).

In many mammals castration increases male lifespan while ovariectomy decreases female lifespan. For example, in studies of rats, castration increased male lifespan (Talbert and Hamilton, 1965; Asdell et al., 1967; Drori and Folman, 1976), but ovariectomy reduced it (Asdell et al., 1967) (though in some of these studies effects did not reach statistical significance). Ovariectomy also reduced survival in mice (Cargill et al., 2003; Benedusi et al., 2015). Castration also extended lifespan in male bank voles (Gipps and Jewell, 1979) and in male feral sheep (Jewell, 1997). Similarly, in humans there is some limited evidence of castration increasing lifespan in men (Hamilton and Mestler, 1969; Min et al., 2012), and more robust evidence that ovariectomy shortens lifespan in women (Rocca et al., 2006; Shoupe et al., 2007; Parker et al., 2009). By contrast gonadectomy increased lifespan in both sexes of domestic cats (Hamilton, 1965; Hamilton et al., 1969; O’Neill et al., 2015) and dogs (particularly in bitches) (Michell, 1999; Hoffman et al., 2013, 2018; O’Neill et al., 2013).

Thus, although germline removal can increase lifespan in both semelparous and iteroparous species, the effects on lifespan are typically far larger and less condition dependent in the former (Table 1), consistent with prevention of reproductive death rather than of the far more modest reproductive costs typical of iteroparous organisms.

**TABLE 1:** Magnitude of increases in lifespan after gonadectomy or behavioral interventions that prevent reproductive death.

	**Lifespan^a^**						
**Species, genotype**	**Sex**	**Intervention**	**Conditions, strain**	**Control**	**Treated**	**% change**	**References**
***C. elegans***							
+	Hermaphrodite	Germline ablation (laser)	20°C, agar plates	19.4 d	31.8 d	+63.9	Hsin and Kenyon, 1999
*daf-2(e1370)*	Hermaphrodite	Germline ablation (laser)	20°C, agar plates	43.2 d	75.7 d	+75.2	Hsin and Kenyon, 1999
+	Hermaphrodite	Germline ablation (laser)	20°C, monoxenic liquid	16.8 d	35.0 d	+108	McCulloch, 2003
*daf-2(e1368), daf-2 RNAi*	Hermaphrodite	Germline ablation (laser)	20°C, agar plates	51.0 d	124.1 d	+143	Arantes-Oliveira et al., 2003
***Caenorhabditis* species**							
*C. elegans*	Hermaphrodite	Germline ablation (laser)	20°C, agar plates	16.7 d	35 d	+109.4	Kern et al., 2020
*C. inopinata*	Female	Germline ablation (laser)	20°C, agar plates	23.5 d	30.7 d	+30.6	Kern et al., 2020
*C. tropicalis*	Hermaphrodite	Germline ablation (laser)	20°C, agar plates	18.8 d	35.9 d	+91	Kern et al., 2020
*C. wallacei*	Female	Germline ablation (laser)	20°C, agar plates	28.7 d	33.9 d	+18.5	Kern et al., 2020
*C. briggsae*	Hermaphrodite	Germline ablation (laser)	20°C, agar plates	17.1 d	31 d	+81.5	Kern et al., 2020
*C. nigoni*	Female	Germline ablation (laser)	20°C, agar plates	29.7 d	34 d	+14.5	Kern et al., 2020
***Pristionchus* species**							
*P. pacificus*	Hermaphrodite	Germline ablation (laser)	20°C, agar plates	24.7 d	40.5 d	+64	Kern et al., 2020
*P. exspectatus*	Female	Germline ablation (laser)	20°C, agar plates	43.1 d	44.3 d	+2.7	Kern et al., 2020
**Semelparous (with reproductive death)**							
*Glycine max* (soy bean)	Monoecious	Flower removal		119 d	179 d	+50.4	Leopold et al., 1959
*O. hummelincki* (octopus)	Female	Optic gland removal		51 d	277 d	+443	Wodinsky, 1977
*A. anguila* (eel)	Unknown	Prevention of sea run	Fresh water	*9 y* ^b^	55 y	+511	Tesch, 1977
*A. anguila* (eel)	Unknown	Prevention of sea run	Fresh water	*9 y* ^b^	88 y	+877	Vladykov, 1956
*O. nerka* (salmon)	Both sexes	Castration		4.8 y	8.5 y	+77.0	Robertson, 1961
*A. stuarti* (marsupial)	Male	Lab capture prior to mating		1 y	3 y	+200	Olsen, 1971
**Iteroparous**							
*D. subobscura*	Female	*grandchildless* mutation	20°C, virgin	58.7 d^c^	67.6 d^c^	+15.1^d^	Maynard Smith, 1958
*D. melanogaster*	Female	*germ cell-less* mutation	25°C, virgin	*44 d*	*38 d*	−13.6	Barnes et al., 2006
*D. melanogaster*	Female	*tudor* mutation	25°C, virgin	*71 d*	*57 d*	−19.7	Barnes et al., 2006
*D. melanogaster*	Female	*bag of marbles* over-expression	25°C	*32,28 d*	*42 d*	+31.3, 50.0	Flatt et al., 2008
*D. melanogaster*	Male	*bag of marbles* over-expression	25°C	*38,36 d*	*46 d*	+21.0, 27.8	Flatt et al., 2008
*R. microptera* (grasshopper)	Female	Ovariectomy	28°C	*167 d*	*205 d*	+22.7	Hatle et al., 2008
*R. microptera* (grasshopper)	Female	Ovariectomy	32°C, 24°C	*245 d*	*285 d*	+16.3	Drewry et al., 2011
*M. musculus* (mouse)	Female	Ovariectomy before puberty	CBA/J	599 d	540 d	−9.8	Cargill et al., 2003
*R. norwegicus* (rat)	Male	Castration at birth	Inbred Lewis	454 d	521 d	+14.7	Talbert and Hamilton, 1965
*R. norwegicus* (rat)	Male	Castration just before puberty	Osborne-Mendel Yale	615 d	651 d	+5.8	Asdell et al., 1967
*R. norwegicus* (rat)	Female	Ovariectomy just before puberty	Osborne-Mendel Yale	742 d	669 d	−9.8	Asdell et al., 1967
*R. norwegicus* (rat)	Male	Castration just before puberty	Norway albino	727 d	817 d	+21.7	Drori and Folman, 1976
*F. catus* (cat)	Male	Castration		4.9 y	8.2 y	+67.3	Hamilton et al., 1969
*F. catus* (cat)	Female	Spayed		6.8 y	8.4 y	+23.5	Hamilton et al., 1969
*F. catus* (cat)	Both sexes	Gonadectomy		*11.0 y*	*15.0 y*	+36.3	O’Neill et al., 2015
*C. lupus familiaris* (dog)	Both sexes	Gonadectomy		7.9 y	9.4 y	+18.9	Hoffman et al., 2013
*H. sapiens*	Male	Castration		*55.7 y*	*69.3 y*	+24.4	Hamilton and Mestler, 1969
*H. sapiens*	Female	Oophorectomy		*65.2 y*	*65.2 y*	+0	Hamilton and Mestler, 1969
*H. sapiens*	Male	Castration		50.9 y	70.0 y	+37.5	Min et al., 2012
*H. sapiens*	Male	Castration		55.6 y	70.0 y	+25.8	Min et al., 2012

*^a^ Mean lifespan (median lifespan, italics; maximum lifespan, underlined). d, days. y, years.*

*^b^ Eels normally live 6–12 y; the median value is taken here.*

*^c^ Life expectancy at age 10 days.*

*^d^ It might be significant that the strain of D. subobscura used in this study mated only once, in contrast to D. melanogaster which can remate multiple times (Partridge and Sibly, 1991). Also included here are behavioral interventions that prevent reproductive death. It is notable that the magnitude of reported experimentally induced increases in lifespan, expressed in terms of proportional increase in lifespan, are generally greater in semelparous than iteroparous organisms.*

### Suppression of Reproductive Death by Germline Ablation in *C. elegans*

Could germline ablation in *C. elegans* hermaphrodites extend lifespan by preventing reproductive death? Supporting this, intestinal atrophy is suppressed by germline removal (Ezcurra et al., 2018; Kern et al., 2020). Moreover, the DAF-16/FOXO transcription factor is required for both life extension (Hsin and Kenyon, 1999) and suppression of intestinal atrophy (Ezcurra et al., 2018) by germline removal.

The striking senescent changes in anatomy seen in hermaphrodites are largely absent from males (de la Guardia et al., 2016; Ezcurra et al., 2018), suggesting that they do not undergo reproductive death. Consistent with this, a study of individually cultured nematodes in monoxenic liquid culture found that germline ablation by laser microsurgery increased lifespan in wild-type hermaphrodites but not males (McCulloch, 2003). Moreover, individually cultured wild-type males are longer lived than hermaphrodites (Gems and Riddle, 2000; McCulloch and Gems, 2007).

We recently examined the pattern of senescent pathology in two additional *Caenorhabditis* species that are, like *C. elegans*, androdioecious (with hermaphrodites and males), *C. briggsae* and *C. tropicalis*, and found them to be similar to *C. elegans*, suggesting the occurrence of reproductive death in these species too (Kern et al., 2020). The majority of *Caenorhabditis* species are gonochoristic (with females and males), and *C. elegans*, *C. briggsae* and *C. tropicalis* represent three independent occurrences of the evolution of androdioecy (Kiontke et al., 2011). Gonochoristic sibling species of these three androdioecious species are, respectively, *C. inopinata*, *C. nigoni*, and *C. wallacei*. Notably, in females (unmated) of these three species the senescent degeneration seen in hermaphrodites does not occur. Moreover, for all three sibling species pairs, hermaphrodites vent yolk and lay unfertilized oocytes, while females do not. However, senescent degeneration was seen in females after mating (Kern et al., 2020). Taken together, these results suggest that after the appearance of hermaphroditism in each instance, reproductive death evolved from being facultative (mating induced) to constitutive. A possible adaptive significance of this change is that females but not hermaphrodites need to await an encounter with a male before commencing reproduction.

Semelparity in *C. elegans* implies a cost of reproduction. It was previously noted that prevention of self-fertilization by means of mutations that impair sperm function does not increase lifespan (Klass, 1977; Kenyon et al., 1993); i.e., the effort of egg production, fertilization and egg laying does not shorten life. This implies that the costly lactational program is active and generates life-shortening pathology whether or not fertilization takes place.

The occurrence of constitutive reproductive death in *Caenorhabditis* hermaphrodites but not females is supported by several further observations. First, for all three sibling species pairs, the females (unmated) are longer lived than the hermaphrodites (Amrit et al., 2010; Kern et al., 2020). In the case of *C. elegans* and its sibling species *C. inopinata*, the latter is longer lived only when the two species are compared in the presence of antibiotics, suggesting greater susceptibility of *C. inopinata* to life-shortening infection by the bacterial food source (Woodruff et al., 2019; Kern et al., 2020).

**FIGURE 6:**
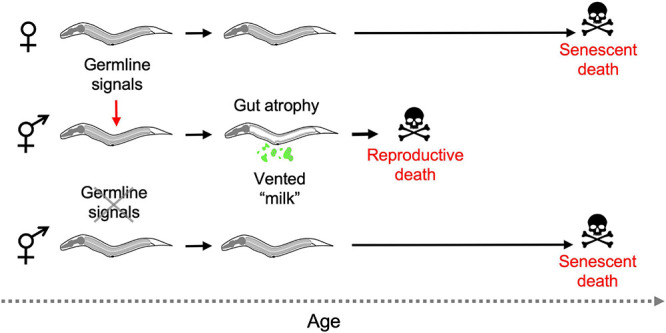
Aging and death in *Caenorhabditis* females and hermaphrodites (simplified working model). In the absence of mating only hermaphrodites exhibit reproductive death, and this is triggered during reproductive maturation by signals from the germline. Removal of the germline by laser microsurgery blocks reproductive death, and markedly extends lifespan in hermaphrodites, removing the difference in lifespan between hermaphrodites and females. Germline ablation only modestly increases female lifespan (not depicted) (Kern et al., 2020).

Combining several of these observations suggests the following scenario: that hermaphrodites but not females undergo reproductive death constitutively, triggered by signals from the germline, leading to shorter lifespan in hermaphrodites. Consistent with this model, germline ablation causes large increases in lifespan in hermaphrodites but not females (Table 1), and abrogates the greater lifespan of females. Moreover, germline ablation suppresses intestinal atrophy in all hermaphroditic species (Kern et al., 2020; see Figure 6 for schematic summary).

Taken together, these observations imply that extension of lifespan by germline ablation in *C. elegans* is due to suppression of semelparous reproductive death, as seen e.g., in Pacific salmon.

### Does Reduced Insulin/IGF-1 Signaling Suppress Reproductive Death?

While the discovery of single gene mutations that alter lifespan in *C. elegans* was important, what generated particular excitement was the large magnitude of increases in lifespan observed, particularly from reductions in insulin/IGF-1 signaling (IIS). The largest effects have been observed in mutants defective in the *daf-2* insulin/IGF-1 receptor and the *age-1* phosphatidyinositol 3-kinase (PI3K) catalytic subunit (Kenyon, 2010) with up to 10-fold increases in mean and maximum lifespan recorded (Ayyadevara et al., 2008). Could these large effects on lifespan reflect suppression of reproductive death, at least in part?

There is some evidence that IIS promotes reproductive death. Mutation of *daf-2* can suppress the dramatic morphological changes accompanying *C. elegans* hermaphrodite senescence (Garigan et al., 2002; Luo et al., 2010; McGee et al., 2012; Ezcurra et al., 2018). IIS also promotes vitellogenesis (Depina et al., 2011; Ezcurra et al., 2018) and venting of yolk milk and laying of yolk-replete oocytes (Gems et al., 1998; Kern et al., 2020). As already mentioned, effects on lifespan of both *daf-2* and germline removal require the DAF-16 FOXO transcription factor (Kenyon et al., 1993; Hsin and Kenyon, 1999) and in both cases its action in the intestine is important (Lin et al., 2001; Libina et al., 2003).

But other observations argue against the idea that reduced IIS extends lifespan simply by blocking reproductive death. First, germline ablation increases lifespan in *daf-2* mutants, seemingly more so than in wild type (+ ∼140% vs. + ∼60%) (Hsin and Kenyon, 1999; Arantes-Oliveira et al., 2002). Second, mutation of *daf-2* increases lifespan in males (Gems and Riddle, 2000; McCulloch and Gems, 2007; Hotzi et al., 2018), though they appear not to exhibit reproductive death. Thus, the relationship of IIS to germline signaling on the one hand and reproductive death on the other remains to be resolved. One possibility is that the two pathways act to some extent in parallel to promote reproductive death, while IIS also impacts lifespan via additional pathway-specific mechanisms, e.g., related to its role in dauer diapause (Kenyon et al., 1993), or to adaptive death (see below).

## A Continuum Between Semelparous and Iteroparous Aging

In this review, we have made the case that *C. elegans* undergo semelparous reproductive death; 12 items of evidence supporting this hypothesis are listed in Table 2.

**TABLE 2:** Features of *C. elegans* consistent with semelparous reproductive death.

(1) *C. elegans* hermaphrodites exhibit early, massive pathology affecting organs linked to reproduction (Garigan et al., 2002; Herndon et al., 2002; Ezcurra et al., 2018).
(2) Gut-to-yolk biomass conversion appears to be part of a suicidal reproductive effort that promotes fitness by feeding trophallactic fluid to larval kin (Kern et al., 2021).
(3) Blocking hermaphrodite reproductive maturation (e.g., by germline ablation) suppresses development of such pathologies, and leads to increases in lifespan of a large magnitude (Hsin and Kenyon, 1999; Ezcurra et al., 2018; Kern et al., 2020).
(4) Germline removal in wild-type males, which do not exhibit semelparity-like pathology, does not increase lifespan (McCulloch, 2003).
(5) *Caenorhabditis* hermaphrodites, which exhibit senescent transformation, are shorter lived than (unmated) *Caenorhabditis* females, which do not, consistent with reproductive death in the former only (Kern et al., 2020).
(6) *Caenorhabditis* hermaphrodites vent yolk and lay unfertilized oocytes in large numbers, while *Caenorhabditis* females do not (Kern et al., 2020).
(7) Germline removal in unmated *Caenorhabditis* females, which do not exhibit semelparity-like pathology, produces much smaller increases in lifespan than in *Caenorhabditis* hermaphrodites (Kern et al., 2020).
(8) Germline removal removes the difference in lifespan between *Caenorhabditis* females and hermaphrodites (Kern et al., 2020).
(9) *C. elegans* senescent transformation involves source-to-sink type resource remobilization, as seen in semelparous animals and plants (Ezcurra et al., 2018; Kern et al., 2021).
(10) Autophagic processes that enable biomass conversion and resource remobilization contribute to senescent pathogenesis in *C. elegans* as in semelparous organisms (particularly plants) (Ezcurra et al., 2018; Benedetto and Gems, 2019).
(11) Semelparous senescence occurs earlier in cell compartments or organs that are non-essential for survival and behavior (e.g., the *C. elegans* intestine) (Ezcurra et al., 2018) than in essential organs (e.g., the *C. elegans* nervous system) (Herndon et al., 2002).
(12) Semelparous species often have an extended pre-reproductive stage, followed by a very brief reproductive stage (c.f. the dauer stage in *C. elegans*) (Klass and Hirsh, 1976).

### Is *C. elegans* a Good Model Organism for Understanding Aging?

Caleb Finch said of semelparous dasyurid marsupials: “Their escape from ‘natural death’ under optimum conditions and their capacity to more than double their natural lifespan caution against overemphasizing lifespan and mortality rates as a basic index of cellular ‘aging’.” (Finch, 1990b, p. 95). Is this warning also applicable to *C. elegans*? If *C. elegans* is semelparous, such that the mechanisms controlling its lifespan are more akin to those in monocarpic plants than in humans, what does this mean for its use as a model organism for studying aging? A great deal of research has been carried out on *C. elegans* aging during the last 40 years; a PubMed search conducted on 20th July 2021 for articles including the terms “elegans” and “aging” identified 4,351 items. Are these studies in fact largely about reproductive death rather than aging?

For *C. elegans* researchers: don’t panic. In the remainder of this essay, we propose a new perspective according to which *C. elegans* is a good model system for studying aging, despite its semelparity. Our key points are as follows. We have argued that *C. elegans* exhibits rapid senescence triggered by sexual maturation and coupled to reproductive effort, as seen in many other semelparous organisms. We postulate: (1) that this form of senescence involves exaggerated versions of mechanisms that are operative in iteroparous organisms, from which they evolved. (2) That such regulated mechanisms of senescence have a much larger effect on lifespan in semelparous organisms than iteroparous organisms. (3) That if such regulated mechanisms are blocked, pathologies that then become life limiting involve a wider spectrum of etiologies—both programmatic [e.g., involving antagonistic pleiotropy (AP) enacted in diverse ways] and stochastic (e.g., molecular damage accumulation, mechanical senescence). According to this view, a virtue of *C. elegans* is that one major form of senescent etiology (programmatic) plays a predominant role in aging, making it more experimentally tractable. This is also an argument for the potential value to understanding animal aging of studying senescence in other semelparous species, including plant models such as *A. thaliana*.

### A Continuum of Semelparity and Iteroparity

Mechanisms in sexual maturation-triggered reproductive death are likely to be related to the subtler mechanisms operative in iteroparous species, consistent with the existing continuum between iteroparity and semelparity (Hughes, 2017). A plausible scenario is that semelparous etiologies evolved by amplification of mechanisms operative in iteroparous ancestors. This resulted in exaggerated and life-limiting senescent pathologies resulting from relatively simple causes. If this were true then semelparous vertebrates should show age-related diseases similar to those seen in iteroparous ones. In fact, this is the case in Pacific salmon, one of the few semelparous vertebrates in which senescent pathologies have been studied. For example in spawning *O. tshawytscha* the coronary arteries, among others, exhibit endothelial cell hyper-proliferation (Figure 3A; Robertson et al., 1961; Farrell, 2002), reminiscent of human coronary artery disease, though lipid and calcium deposits typical of mammalian atheromas are not seen (Robertson et al., 1961; House and Benditt, 1981). Furthermore, starting at sexual maturity kokanee salmon develop amyloid deposits in multiple regions of the brain, similar to those occurring in Alzheimer’s disease in humans (Figure 3A; Maldonado et al., 2000, 2002). This is one of the few examples of Alzheimer-like cytopathology found in wild vertebrates under natural conditions. The pathology includes extracellular amyloid plaques that are immunoreactive with anti-Aβ_1__–__4__2_ antibodies. The distribution of amyloid deposition is similar to that of glucocorticoid receptors, suggesting that elevated glucocorticoids may cause this Alzheimer-like pathology (Maldonado et al., 2000). Similarly, thymic involution is promoted by sex steroid-induced glucocorticoid production in both spawning salmon and in mammals (Chen et al., 2010). It is also possible that IGF-1 (cf IIS) promotes reproductive death in salmon, e.g., through effects on gonadal development (Allard and Duan, 2011). Notably, many of the pathological changes that occur rapidly in spawning salmon also occur in later life in castrated salmon, i.e., reproductive death resembles accelerated aging (Robertson and Wexler, 1962).

One broad difference between semelparous etiologies and the iteroparous etiologies from which they evolved is that while the former are irreversible the latter can be reversible. For example, intestinal atrophy in adult *C. elegans* hermaphrodites or spawning lampreys appears to be irreversible, whereas loss of muscle during starvation or bone during lactation is reversible (Speakman, 2008). In summary, the nature of the diseases of aging in Pacific salmon supports the existence of a continuum between semelparous and iteroparous species in terms of senescent pathophysiology.

### Quasi-Programs vs. Costly Programs as Ubiquitous Causes of Senescence

As a broad approximation, in terms of primary mechanisms, senescence has been viewed either as a passive process of stochastic damage and breakdown (loss of function), or as an active process driven by late-life effects of gene action (hyperfunction) (Harman, 1956; Blagosklonny, 2006; Gems and Partridge, 2013; Gladyshev, 2013; Shore and Ruvkun, 2013). In semelparous organisms, the mechanisms that give rise to senescent pathogenesis (such as those involving resource reallocation) are clearly active, programmed processes; here pathology is generated as a by-product of functions that promote fitness, but is not itself advantageous (Williams, 1957). In iteroparous organisms (including most mammals) senescent pathologies can result, at least in part, from programmatic mechanisms such as futile quasi-programs.

To understand the relevance of aging in *C. elegans* to that in iteroparous organisms we need to ask: What is the relationship between reproductive death and the quasi-program (hyperfunction) theory? Here the growing understanding of senescent pathophysiology in *C. elegans* is instructive. Several major senescent pathologies, including intestinal atrophy, yolk accumulation and teratoma-like uterine tumors, have been interpreted as resulting from hyperfunction rather than molecular damage, and from run-on type quasi-programs (Herndon et al., 2002; Ezcurra et al., 2018; Wang et al., 2018b). However, the recent discovery that vented yolk promotes larval growth implies that yolk synthesis in sperm-depleted mothers is not, in fact, futile at all, but instead promotes fitness through resource reallocation from mothers to larval kin (Kern et al., 2021).

Thus, late-life yolk production and the intestinal atrophy to which it is coupled does not conform to Blagosklonny’s definition of a quasi-program (futile program continuation). Instead it involves a physiological trade-off where intestinal senescence is a cost. However, in both cases, the mechanisms involved are programmatic, rather than relating to damage and maintenance.

Another difference between these two cases is the relative timing of benefits and costs. In the first account, a program that promotes fitness in early life becomes a harmful quasi-program in later life. By contrast, in the case of intestinal atrophy coupled to yolk production, benefit and harm are generated simultaneously.

**FIGURE 7:**
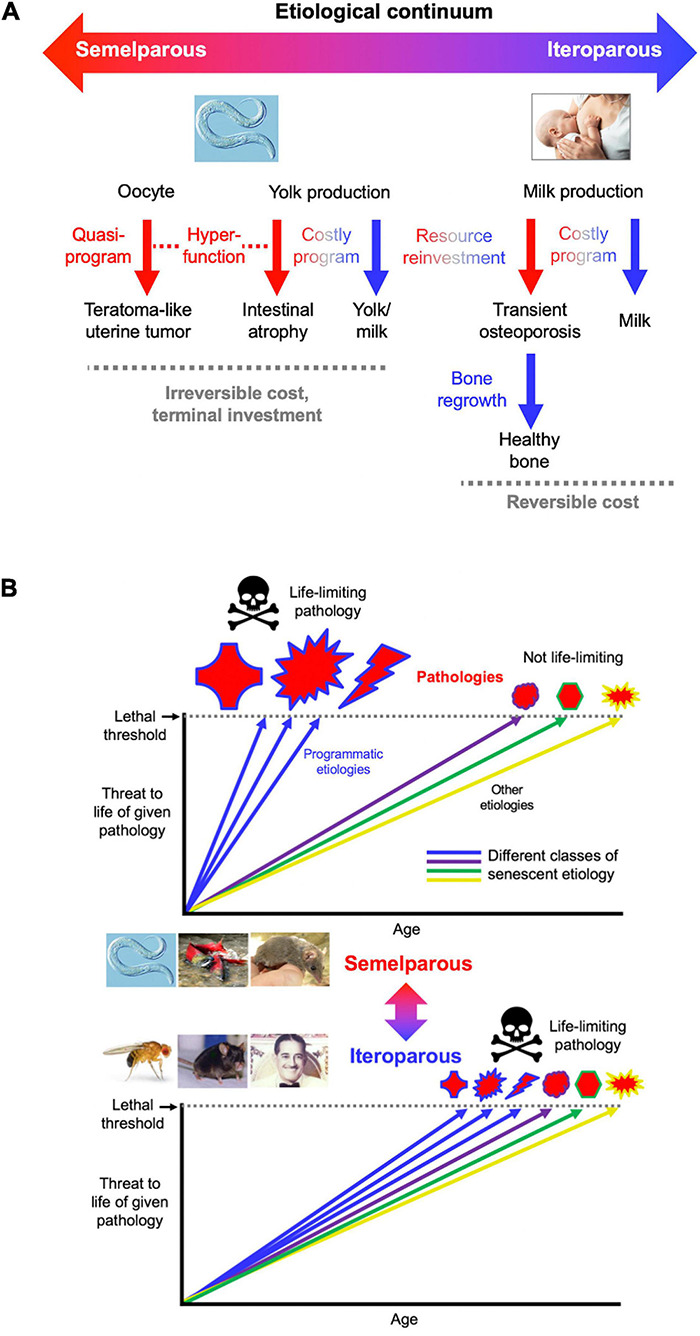
Conceptual models of aging in semelparous and iteroparous organisms. **(A)** Programmatic mechanisms of aging in semelparous and iteroparous organisms. These include costly programs and quasi-programs. A broad prediction is that costly programs contribute more to disease during reproductive death, and quasi-programs more in iteroparous aging. **(B)** Difference in senescent pathogenesis in semelparous and iteroparous organisms. The figure shows the degree of harmfulness of a range of pathologies with different types of etiology (indicated by different colors). Top, reproductive death. Here exaggeration of programmatic mechanisms leads to rapid development of gross pathologies leading to death. Bottom, typical animal senescence (iteroparous species). Here many more types of etiology contribute to life-limiting pathology, to which programmatic etiologies contribute to some degree, and senescence is more multifactorial. Preventing programmatic pathophysiology that causes reproductive death causes very large increases in lifespan, giving a false impression that the entire aging process has been suppressed. All images reproduced with permission.

Insofar as the term *program* implies complex function *and* promotion of fitness (Lohr et al., 2019), *C. elegans* intestinal resource reallocation may be referred to as a *costly program*. By contrast, development of uterine teratomas is the result of a quasi-program (Wang et al., 2018a, b; Figure 7A), since a fitness benefit from having tumors is difficult to envisage.

To create an integrated conceptual framework we propose the following new account: that in both cases (quasi-programs and costly programs), pathology results primarily from hyperfunction rather than loss of function. In costly programs hyperfunction exists with respect to the pathology (e.g., intestinal atrophy) but not the benefit (yolk milk production) (Figure 7A). Similarly, the process of N remobilization from leaves is hyperfunctional as far as leaf health is concerned, but not seed provisioning. Thus, precise use of the term hyperfunction requires reference to the entity that it injures (cell, tissue, organ, organism).

According to this account, in iteroparous organisms resource reallocation can involve costly programs where the debts can be repaid, as in lactation-associated bone loss or starvation-induced muscle atrophy. Thus, *C. elegans* reproductive death, like mammalian aging, involves both quasi-programs and costly programs (Figure 7A). Understanding *C. elegans* aging should therefore provide fundamental insights into the pathophysiology of human senescence.

### Neuroendocrine Promotion of Semelparous and Iteroparous Aging

Further evidence of conservation of mechanisms of aging between *C. elegans* and iteroparous species (e.g., *Drosophila*, rodents) is that insulin/IGF-1 and mTOR signaling promote aging in both (Kenyon, 2010; Weichhart, 2018). However, in *C. elegans* the magnitude (in relative terms) of life extension resulting from reduced IIS is typically far greater than in iteroparous species; for example, mutational reduction of PI3K increases median lifespan by up to ∼10-fold in *C. elegans* but only ∼1.07- and ∼1.02-fold in *Drosophila* and mice, respectively (Ayyadevara et al., 2008; Slack et al., 2011; Foukas et al., 2013). This is consistent with the idea that programmatic etiologies occur in both semelparous and iteroparous species, are amplified in the former, and promoted by IIS in both. According to this view, although the large magnitude of the effect of IIS on lifespan in *C. elegans* reflects suppression of reproductive death, the etiology of senescent pathology in reproductive death is fundamentally similar to that of some senescent pathologies that contribute to late-life mortality in iteroparous species (including humans) (Figure 7B).

Such effects of IIS on aging are part of a broader neuroendocrine and steroid hormone signaling network affecting growth, reproduction and lifespan in both semelparous and iteroparous organisms (Finch, 1990a; Partridge and Gems, 2002; Gáliková et al., 2011; Atwood et al., 2017; Bartke, 2019). For example, in *C. elegans* sensory neurons exert IIS-mediated effects on lifespan (Apfeld and Kenyon, 1999), and germline effects on lifespan are mediated by steroid signaling (Antebi, 2013). In octopus the optic gland, equivalent to the pituitary gland, promotes vitellogenesis and reproductive death (Wodinsky, 1977). Reproductive death in salmon and *Antechinus* is driven by adrenohypercorticism (Barker et al., 1978; Mcquillan et al., 2003). In amphibians, reptiles and birds, vitellogenesis is promoted by growth hormone (GH) and estrogen (Dolphin et al., 1971; Wallace, 1985). In mammals pituitary GH acts through IGF-1 to promote gonadal growth and reproduction (Chandrashekar et al., 2004) and of course female reproductive function is regulated by estrogen. Taken together this, again, supports the view that reproductive death evolves by exaggeration of mechanisms (here endocrine) operative in iteroparous species.

### Reproductive Death and Adaptive Death

**FIGURE 8:**
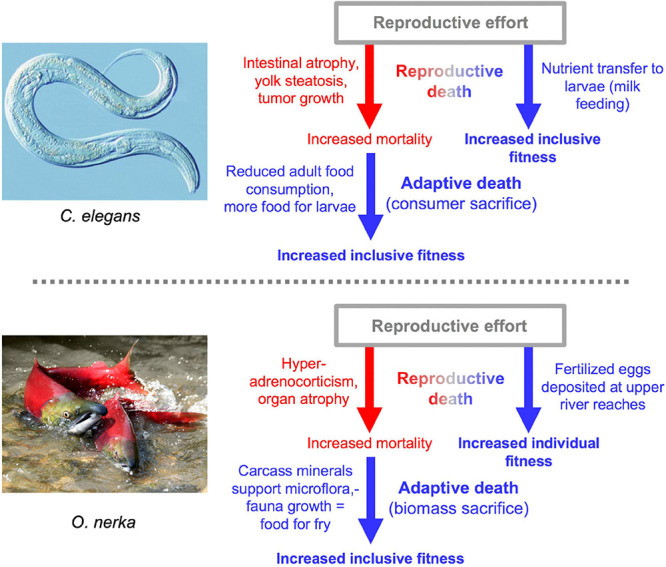
Double death: Reproductive death and adaptive death combine to promote fitness. In both cases there is evidence for the existence of adaptive death, but its existence has not been definitively proven (Galimov and Gems, 2021). The two examples of double death differ in that adaptive death in *C. elegans* involves consumer sacrifice (removing a consumer to increase food availability for kin) while in *O. nerka* it involves biomass sacrifice (dying to facilitate resource remobilization) (Lohr et al., 2019).

Besides semelparous reproductive death and iteroparous senescence, another mode of life-limiting mechanism is programmed adaptive death. Here genetically determined mechanisms that actively cause death have evolved by natural selection because earlier death provides an inclusive fitness benefit, in a manner analogous to programmed cell death in metazoan organisms. Adaptive death is not expected to evolve in organisms with outbred, dispersed populations (e.g., most animal species), but can occur in those existing as compact (viscous) colonies of clonal individuals, such as the yeast *Saccharomyces cerevisiae* and possibly *C. elegans* too (Galimov et al., 2019; Lohr et al., 2019; Galimov and Gems, 2021). Evolutionary theory predicts that adaptive death can more readily evolve in the presence of semelparity, which could explain the apparent presence of both in *C. elegans* and Pacific salmon (Galimov and Gems, 2021; Figure 8).

## Perspectives

This essay presents an altered picture both of *C. elegans* as a model for aging research, and of aging more broadly. These changes imply some gains to the field, but also one grievous loss. The gains include an understanding that *C. elegans* is semelparous, and that the mechanisms involved in semelparous aging are a programmatic subset of those involved in iteroparous aging. This implies that *C. elegans* is an excellent model for studying programmatic mechanisms of senescence in a conveniently exaggerated and relatively pure form. Programmatic mechanisms potentially contribute to many diseases of human aging, for example those promoted by senescent cells, which are at least partly caused by quasi-programs (Blagosklonny, 2006). It also suggests that suppression of such mechanisms could unmask and bring into play more of the determinants of lifespan that are operative in iteroparous species. Recognition of the continuum between mechanisms of semelparous and iteroparous aging also removes a spurious separation between the biology of animal aging and plant senescence; from henceforth, scientists studying plant senescence ought to receive more invitations to biogerontology meetings.

Regarding the loss. Aging is now the main cause of disease and death worldwide, and yet its underlying mechanisms remain unclear. The discovery over three decades ago that single gene mutations can greatly increase lifespan in *C. elegans* (Klass, 1983; Friedman and Johnson, 1988; Kenyon et al., 1993) had extraordinary implications. First, the large increases in lifespan suggested the existence of core mechanisms underlying the entire aging process. Second, they implied that these mechanisms could be manipulated to slow down aging. Third, given that *C. elegans* is a highly tractable model organism, it suggested that it ought to be relatively easy to define these core mechanisms of aging. What is often exciting about studies in model organism biogerontology is the possibility that they bring us closer to a knowledge of these mysterious central mechanisms of aging, whose discovery promised to make possible extraordinary things in terms of slowing human aging and extending lifespan. The interpretations in this essay in some sense explain away the mystique of *C. elegans* life extension. We suggest that these large increases in lifespan could reflect suppression of reproductive death. This involves suppression of grossly exaggerated versions of programmatic mechanisms that are only one cause of aging in iteroparous organisms. More seriously, it also suggests that increases in lifespan achieved in iteroparous organisms may also reflect action on weaker programmatic determinants of senescence that are only a minor subset of the determinants of aging. This would imply relatively limited plasticity in aging in iteroparous organisms. Thus, the new picture that we present is, arguably, more realistic but less magical.

## Data Availability Statement

The original contributions presented in the study are included in the article/supplementary material, further inquiries can be directed to the corresponding author/s.

## Author Contributions

DG wrote the manuscript, with contributions from CK, JN, and ME. All authors contributed to the article and approved the submitted version.

## Conflict of Interest

The authors declare that the research was conducted in the absence of any commercial or financial relationships that could be construed as a potential conflict of interest.

## Publisher’s Note

All claims expressed in this article are solely those of the authors and do not necessarily represent those of their affiliated organizations, or those of the publisher, the editors and the reviewers. Any product that may be evaluated in this article, or claim that may be made by its manufacturer, is not guaranteed or endorsed by the publisher.

## Glossary

***Adaptive death****:* Synonymous with programmed organismal death. Here death of an individual is a selected trait, providing a direct benefit in terms of inclusive or group fitness (Lohr et al., 2019).

***Aging****:* In the context of this article, synonymous with senescence, i.e., the deteriorative changes that become progressively worse with advancing age, leading to multiple pathologies and death. We argue here that reproductive death is a form of rapid aging, since it involves mechanisms that also contribute to iteroparous aging. From this perspective, leaf senescence may be viewed as a form of aging.

***Androdioecious****:* Where adults are male or hermaphrodite (as opposed, in the context of this essay, to male or female).

***Antagonistic pleiotropy****(AP):* Where action of a given gene leads to both fitness benefits and fitness costs. If the latter occur later in life and are therefore subject to weaker selection, such a gene may be favored by natural selection, and promote aging (Williams, 1957).

***Costly program****(New term):* A biological program that simultaneously causes fitness benefits, and costs in terms of pathological changes to tissues or organs where the program is executed. One form of programmatic mechanism involving hyperfunction by which AP causes senescence (cf. quasi-program).

***Demolition engineer principle****(New term****)****:* Dual function of autophagic processes during resource remobilization, to both effect destructive turnover of cellular components, and maintain cellular homeostasis.

***Disposable soma****:* Theory proposing that natural selection favors investment of limited resources into reproduction rather than somatic maintenance, accelerating damage accumulation and, therefore, senescence (Kirkwood, 1977).

***Fitness****:* The measure of how well a given species is able to survive and reproduce.

***Gonochoristic****:* Where adults are male or female (as opposed, in the context of this essay, to male or hermaphrodite).

***Hyperfunction****:* Where wild-type gene function actively leads to senescent pathology, as opposed to passive random damage or wear and tear (Blagosklonny, 2006).

***Iteroparous****:* Where multiple reproductive cycles can occur over the course of a lifetime.

***Programmed aging****:* Senescence caused by a relatively ordered series of biological processes that promotes fitness via inclusive fitness or group fitness.

***Programmatic aging****:* Where complex biological processes contributes to senescence, but not necessarily to fitness (cf. quasi-programs, costly programs).

***Quasi-programmed aging****:* Senescence caused by a relatively ordered series of biological processes that does not promote fitness; may occur due to futile run-on of wild-type programs that promote fitness earlier in life (Blagosklonny, 2006).

***Reproductive death****:* A form of suicidal reproductive effort found in some semelparous species (e.g., Pacific salmon, monocarpic plants). Here, reproductive maturity triggers the rapid development of lethal pathologies and fast senescence coupled to reproductive success (Finch, 1990b).

***Resource reallocation****:* Where the building blocks of life (e.g., amino acids, lipids, carbohydrates) are transferred from one site (e.g., tissue, organ) to another, in a manner that typically involves breakdown (e.g., autophagic) of source biomass.

***Run-on****:* Futile continuation of gene function or processes in later life, leading to pathology (de la Guardia et al., 2016) (cf. quasi-program).

***Semelparous****:* Organisms with a single reproductive episode before death. Also used to denote semelparity with reproductive death.

***Senescence****:* The overall process of deterioration with age or the resulting pathological condition (not to be confused with *cellular senescence*, which is a particular form of cell cycle arrest affecting some vertebrate cell types). Although *aging* has several meanings, in the biological context it is usually synonymous with senescence.

***Source-to-sink biomass conversion****:* Resource remobilization where autophagic processes break down cellular constituents in one tissue/organ to provide resources for another (cf. costly program).
